# Antibodies Elicited in Response to EBNA-1 May Cross-React with dsDNA

**DOI:** 10.1371/journal.pone.0014488

**Published:** 2011-01-04

**Authors:** Pragya Yadav, Hoa Tran, Roland Ebegbe, Paul Gottlieb, Hui Wei, Rita H. Lewis, Alice Mumbey-Wafula, Atira Kaplan, Elina Kholdarova, Linda Spatz

**Affiliations:** 1 Department of Chemistry, The City College of New York and the Graduate Center of the City University of New York, New York, New York, United States of America; 2 The Ph.D. program in Biochemistry, The City College of New York and the Graduate Center of the City University of New York, New York, New York, United States of America; 3 The Graduate School of Biology, The City College of New York, New York, New York, United States of America; 4 Department of Microbiology and Immunology, Sophie Davis School of Biomedical Education, The City College of New York, New York, New York, United States of America; Instituto Butantan, Brazil

## Abstract

**Background:**

Several genetic and environmental factors have been linked to Systemic Lupus Erythematosus (SLE). One environmental trigger that has a strong association with SLE is the Epstein Barr Virus (EBV). Our laboratory previously demonstrated that BALB/c mice expressing the complete EBNA-1 protein can develop antibodies to double stranded DNA (dsDNA). The present study was undertaken to understand why anti-dsDNA antibodies arise during the immune response to EBNA-1.

**Methodology/Principal Findings:**

In this study, we demonstrated that mouse antibodies elicited in response to EBNA-1 cross-react with dsDNA. First, we showed that adsorption of sera reactive with EBNA-1 and dsDNA, on dsDNA cellulose columns, diminished reactivity with EBNA-1. Next, we generated mononclonal antibodies (MAbs) to EBNA-1 and showed, by several methods, that they also reacted with dsDNA. Examination of two cross-reactive MAbs—3D4, generated in this laboratory, and 0211, a commercial MAb—revealed that 3D4 recognizes the carboxyl region of EBNA-1, while 0211 recognizes both the amino and carboxyl regions. In addition, 0211 binds moderately well to the ribonucleoprotein, Sm, which has been reported by others to elicit a cross-reactive response with EBNA-1, while 3D4 binds only weakly to Sm. This suggests that the epitope in the carboxyl region may be more important for cross-reactivity with dsDNA while the epitope in the amino region may be more important for cross-reactivity with Sm.

**Conclusions/Significance:**

In conclusion, our results demonstrate that antibodies to the EBNA-1 protein cross-react with dsDNA. This study is significant because it demonstrates a direct link between the viral antigen and the development of anti-dsDNA antibodies, which are the hallmark of SLE. Furthermore, it illustrates the crucial need to identify the epitopes in EBNA-1 responsible for this cross-reactivity so that therapeutic strategies can be designed to mask these regions from the immune system following EBV exposure.

## Introduction

Systemic Lupus Erythematosus (SLE) is a chronic autoimmune disease characterized by the production of antibodies to double stranded DNA (dsDNA) and ribonucleoproteins. The etiology of SLE is unknown, although genetic and environmental causes have been implicated. Several viruses have been linked to SLE, however, the strongest association has been made with the Epstein-Barr virus (EBV). EBV is a lymphotropic, dsDNA herpes virus that infects 90–95% of adults in the United States [1]. Despite this high incidence of infection, only a small subset of infected individuals will develop SLE [2]. Epidemiological studies have demonstrated a higher incidence of EBV infection and higher titers of antibodies to EBV in both young and adult lupus patients relative to healthy individuals. James et al., observed seroconversion (development of IgG antibodies to EBV viral capsid antigen) in 99% of adolescent SLE patients compared to 70% of healthy adolescents and 72% of adolescents with other rheumatic diseases [3]. In addition, they observed by PCR analysis, the presence of EBV DNA in lymphocytes of 100% of SLE patients tested, compared to 72% of controls. McClain et.al. observed that antibodies to a major EBV nuclear antigen, EBNA-1, which is continuously expressed in latently infected B cells, arose in all pediatric SLE patients examined compared to only 69% of healthy pediatric controls [4].

EBNA-1 is a DNA binding protein that maintains replication of the EBV genome within infected cells. It is also required for maintaining viral latency. Several studies suggest that exposure to EBNA-1 following EBV infection, can lead to an autoimmune response in some individuals, which may play a role in SLE disease etiology. It has been reported that antibodies to epitopes on EBNA-1 cross-react with epitopes on Sm, a ribonucleoprotein complex consisting of a core of polypeptides (B/B′, D, E, F, G) [5], [6]. Sabbatini et al. demonstrated that antibodies to Sm D could be generated in mice immunized with a Gly-Arg rich peptide derived from the amino terminal end of EBNA-1 [7]. James et al revealed that antibodies to Sm B/B′ could be elicited in rabbits and mice following immunization with a proline rich peptide in the carboxyl end of EBNA-1 (PPPGRRP) that has homology to a proline rich region (PPPGMRPP) found in Sm [8]. In addition, they observed that some animals subsequently developed antibodies to dsDNA , which they hypothesized arose as a consequence of epitope spreading, although this was not proven. More recently, Poole et al showed that rabbits and mice injected with the proline rich peptide of EBNA-1, subsequently develop antibodies to U1 ribonucleoproteins, RNP A and RNP C as a consequence of epitope spreading [9].

Our laboratory previously reported, that BALB/c mice immunized with an EBNA-1 expression vector that expressed either the entire EBNA-1 protein or EBNA-1 lacking the Gly-Ala repeat, developed antibodies to dsDNA as well as to Sm [10]. It was assumed that the antibodies to Sm arose because of cross-reactivity with EBNA-1 as previously reported, however, the basis for the anti-dsDNA response was unknown. The present study was undertaken to address this issue. Our results strikingly reveal that many antibodies elicited in response to EBNA-1 actually cross-react with dsDNA.

## Results

### Mice injected with purified recombinant EBNA-1 protein develop antibodies to dsDNA

We were interested in determining how anti-dsDNA antibodies could arise in mice that develop anti-EBNA-1 antibodies upon exposure to EBNA-1 protein. In our previous study, we generated an anti-EBNA-1 response in mice by injecting them with an EBNA-1 expression vector. However, not all mice developed anti-EBNA-1 antibodies, presumably because they did not all express an adequate concentration of the EBNA-1 protein [10]. In the present study, in order to examine the EBNA-1 response, we decided to inject mice with purified recombinant EBNA-1 protein (rEBNA-1) rather than the EBNA-1 expression vector. EBNA-1 protein used for injections was prepared in our laboratory from a baculovirus vector obtained from Lori Frappier (McMaster University, Ontario, Canada). The rEBNA-1 protein encoded by this vector lacks most of the Gly-Ala repeat. It has been shown that the Gly-Ala repeat is not required for the replication, transactivation or segregation function of EBNA-1, although, it does enable EBNA-1 to escape detection by cytotoxic CD8^+^ T cells [11], [12]. The MW of the rEBNA-1 protein lacking the Gly-Ala repeat is approximately 52Kda.

**Figure 1:**
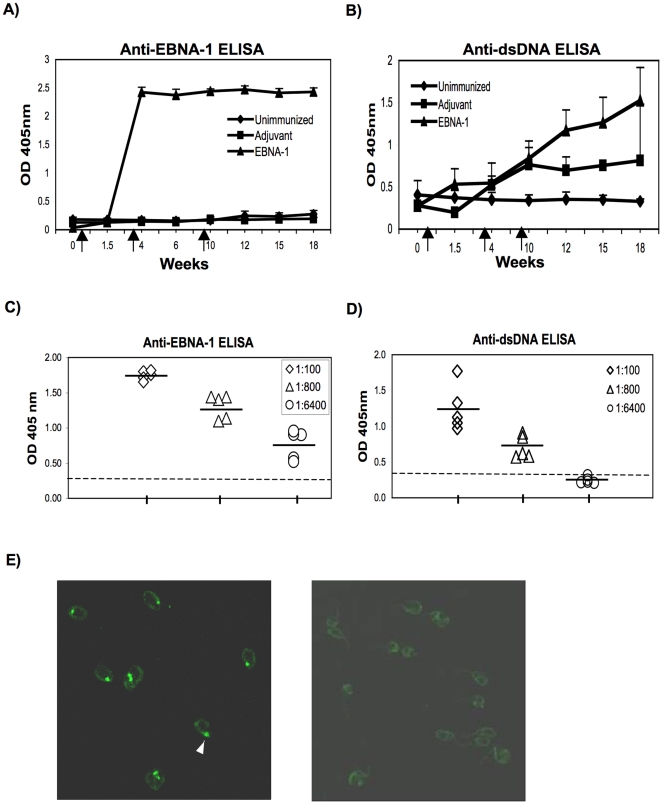
rEBNA-1 injected mice develop antibodies to EBNA-1 and dsDNA. Mice were unimmunized or injected ip with rEBNA-1 emulsified in CFA or CFA alone and boosted with rEBNA-1 emulsified in IFA or IFA alone (arrows indicate week of initial injection and boosts). Mice were bled at weeks 1.5, 4, 6, 10, 12, 15, and 18 and sera tested by ELISA for antibody to EBNA-1 (A) and dsDNA (B). Results are the average of 5 mice in each group. Standard deviations are indicated. (C and D) Eight fold serial dilutions of week 12 sera from all 5 rEBNA-1 injected mice were tested by ELISA for antibody titers to EBNA-1 (C) and dsDNA (D). Dotted line represents 3 standard deviations above the mean absorption of sera from week 12 uninjected control mice. (E) Serum from a week 12 mouse injected with rEBNA-1 (left panel) or adjuvant only (right panel) was diluted 1∶50 and used to immunostain Crithidia luciliae slides. Immunostaining of the dsDNA in the kinetoplast of Crithidia luciliae is observed (left panel) as indicated by arrow. Results are representative of 3 rEBNA-1 and 3 adjuvant only, injected mice.

Five, 6 week old, female, BALB/c mice were injected with 50 µg of rEBNA-1 protein in CFA and were boosted 2 times at weeks 3 and 9 with 25 µg of rEBNA-1 in IFA. Five age and sex matched control BALB/c mice were immunized with CFA alone and boosted with IFA and 5 mice were used as uninjected age matched controls. We observed that all 5 mice injected with rEBNA-1 developed IgG antibodies to EBNA-1 within the first 3 weeks (Figure 1A). In addition, mice developed antibodies to dsDNA, although, the kinetics of the anti-dsDNA response lagged behind that of the anti-EBNA-1 response suggesting that anti-dsDNA antibodies may have developed over time as a consequence of epitope spreading or somatic mutation (Figure 1B). Some mice immunized with adjuvant only, also developed antibodies to dsDNA but with the exception of one mouse, their levels of anti-dsDNA antibody were never as high as that of mice injected with rEBNA-1 in adjuvant. Intraperitoneal delivery of CFA has been shown by others to elicit the production of autoantibodies in mice, including the production of anti-DNA antibodies [13]. It is extremely unlikely that the anti-dsDNA response in rEBNA-1 injected mice was due primarily to adjuvant, as our previous DNA based inoculation studies using EBNA-1 expression vectors in the absence of adjuvant, also elicited the production of anti-dsDNA antibodies [10].

Week 12 sera from all 5 rEBNA-1 injected mice were serially diluted and tested for binding to EBNA-1 (Figure 1C) and dsDNA (Figure 1D) by ELISA. All rEBNA-1 injected mice developed high titers of antibody to EBNA-1 and dsDNA. However, the anti-EBNA-1 titers were higher than the anti-dsDNA titers suggesting that either the concentration of antibodies to EBNA-1 were higher than the concentration of antibodies to dsDNA or the affinities of the antibodies to EBNA-1 were higher than for dsDNA. At a dilution of 1∶6400, the anti-EBNA-1 response in all mice was greater than 3 standard deviations above the mean of similarly diluted uninjected control mice (dotted line). At a dilution of 1∶800, the anti-dsDNA response was greater than 3 standard deviations above the mean of uninjected control mice (dotted line). No anti-dsDNA response was observed at a dilution of 1∶6400.

To confirm the specificity of the anti-dsDNA response, week 12 sera from mice injected with rEBNA-1 were diluted 1∶50 and used to immunostain Crithidia luciliae slides. The presence of antibody to dsDNA was indicated by binding to the kinetoplast (Figure 1E, left panel). In contrast, sera from adjuvant immunized mice did not reveal kinetoplast binding (Figure 1E, right panel ) indicating that either the anti-DNA antibodies present in these mice were of lower affinity than the anti-dsDNA antibodies obtained from rEBNA-1 injected mice or they were not specific for dsDNA.

**Figure 2:**
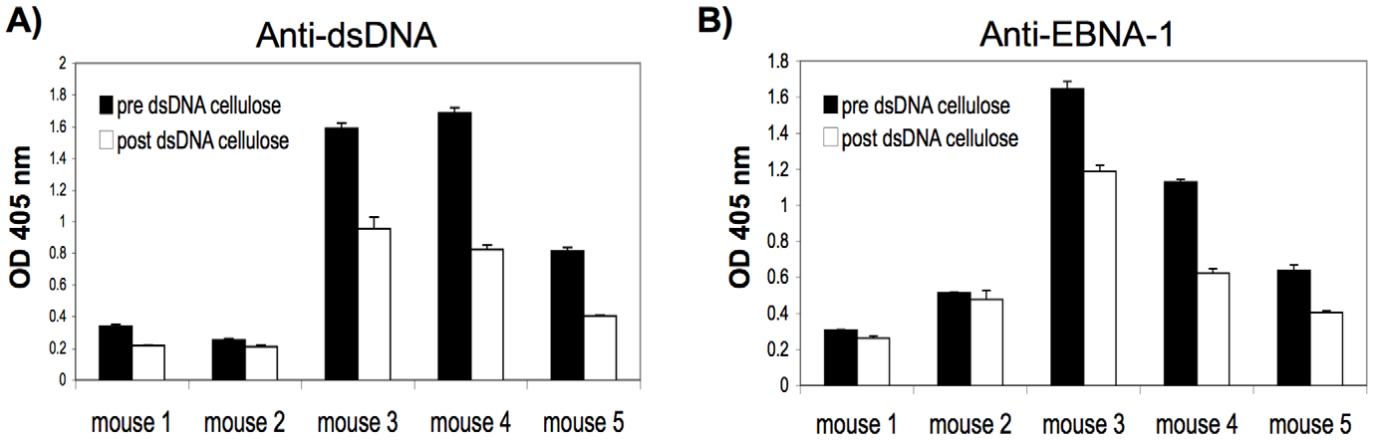
Sera from EBNA-1 injected mice display reduced binding to EBNA-1 following adsorption onto dsDNA cellulose beads. Week 12 sera from all five rEBNA-1 injected mice were adsorbed onto dsDNA cellulose beads and pre and post adsorbed sera were tested by ELISA for binding to dsDNA (A) and EBNA-1 (B). Results represent OD 405 nm values after subtraction of non specific binding to cellulose only beads. Standard deviations of triplicate wells are indicated. Sera were diluted 1∶5000 prior to adsorption. One tailed, unpaired t test was used to compare OD 405 nm before and after adsorption on dsDNA cellulose (t<0.001 for mouse 1,3, 4, and 5 in (A) and t<0 .005 for mouse 1, 3, 4, and 5 in (B)).

To determine whether any of the antibodies to EBNA-1generated in rEBNA-1 injected mice also cross-reacted with dsDNA, week 12 sera from all 5 EBNA-1 injected mice were adsorbed over dsDNA-cellulose beads to remove dsDNA reactive antibodies and then sera were tested by ELISA to determine if adsorbed sera showed reduced binding to EBNA-1. Loss of antibody in the sera due to non specific sticking to cellulose was determined by adsorbing sera to cellulose only beads. Figure 2, represents the OD 405 nm of anti-dsDNA (A) and anti-EBNA-1 antibody (B), pre and post adsorption onto dsDNA cellulose beads, after the value for non specific binding to cellulose was subtracted. We observed a significant decrease in anti-dsDNA antibody activity following adsorption on dsDNA cellulose beads, in mouse 1, 3, 4, and 5 (p<0.001) (Figure 2A). In a similar trend, we observed a significant decrease in anti-EBNA-1 activity in the sera from mouse 1, 3, 4, and 5, following adsorption on dsDNA cellulose (p<0.005) (Figure 2B). Mouse 2 showed a small decrease in anti-dsDNA and anti-EBNA-1 activity following adsorption on dsDNA cellulose although it was not significant. This is likely because mouse 2 developed a negligible response to dsDNA following injection with rEBNA-1 although the anti-EBNA-1 response was significant. The observation that a reduction of anti-dsDNA antibody on a dsDNA cellulose column, led to a parallel reduction of anti-EBNA-1 activity, suggests that anti-EBNA-1 antibody in the sera of some rEBNA-1 injected mice, cross-reacts with dsDNA.

Since week 4 rEBNA-1 injected mice displayed a significant delay in the development of a high titer anti-dsDNA but not anti-EBNA-1 response (Figure 1B), we wanted to examine whether there was any evidence of cross-reactivity to dsDNA in week 4 sera. We therefore adsorbed week 4 sera over dsDNA cellulose beads. Interestingly, we also observed some reduction in binding to dsDNA and EBNA-1 following adsorption on dsDNA cellulose (data not shown) suggesting that the cross-reactive response arose early. However, since the anti-dsDNA response was much weaker at week 4 than week 12, epitope spreading may have played a role in refining the cross-reactive response over time.

### Generation of monoclonal antibodies to EBNA- 1 that cross-react with dsDNA

Although, adsorption studies suggested that antibodies to EBNA-1 generated in rEBNA-1 injected mice cross-creacted with dsDNA they did not prove this. To confirm these results, we therefore generated monoclonal anti-EBNA-1 antibodies from rEBNA-1 injected mice and tested these antibodies for binding to dsDNA by ELISA. To generate monoclonal antibodies to EBNA-1, splenocytes from rEBNA-1 injected BALB/c mice containing serum IgG antibodies to both EBNA-1 and dsDNA, were fused to NSO cells. Hybridoma supernatants were screened by ELISA for IgG antibodies to EBNA-1 and dsDNA. In an initial screen of one fusion, we observed that the majority of clones that tested positive for antibody reactivity to EBNA-1 were also positive for reactivity to dsDNA (10 out of 14 or 71%). Seven clones that were positive for antibodies to EBNA-1 were subcloned two times to insure clonality. Supernatants from these hybridomas were then tested for antibodies to EBNA-1 and dsDNA. In addition, they were tested for the presence of antibodies to the blocking agent Bovine Serum Albumin (BSA). One of the subclones, 3F3, secreted antibody specific for EBNA-1 only (Table 1). Three subclones, secreted monoclonal antibody that reacted strongly with both EBNA-1 and dsDNA and did not bind BSA. Subclone 3D4 is representative of this group (Table 1). Three other subclones, represented by 9G3, secreted antibody that bound not only to EBNA-1 and dsDNA but BSA as well (Table 1).

**Table 1:** Reactivity of representative monoclonal antibodies to EBNA-1, dsDNA, and BSA.

MAbs	anti-EBNA-1	anti-dsDNA	anti-BSA	Source
**3D4**	+++	+++	−	this study
**9G3**	+	+	+	this study
**3F3**	++	−	−	this study
**0211**	++	++	−	Commercial MAb Pierce, Rockford, IL

+++ strong binding.

++ moderate binding.

+ weak binding.

In the present study we chose to focus on 3D4 because it is an IgG antibody that binds strongly to dsDNA, which is characteristic of many pathogenic IgG anti-dsDNA antibodies that arise in SLE. In addition, it's strong binding to both EBNA-1 and dsDNA made it a good candidate for studying the basis of this cross-reactive response. The other antibodies such as 9G3 that also reacted with BSA (and casein), were not further characterized in this study because of concern that this would lead to non specific binding in ELISAs and Western blots; assays which require these blocking reagents. In addition these antibodies displayed a much weaker affinity for EBNA-1 and dsDNA making them less desirable to use in our initial attempt to identify epitopes in EBNA-1 that play a role in cross-reactivity to dsDNA.

**Figure 3:**
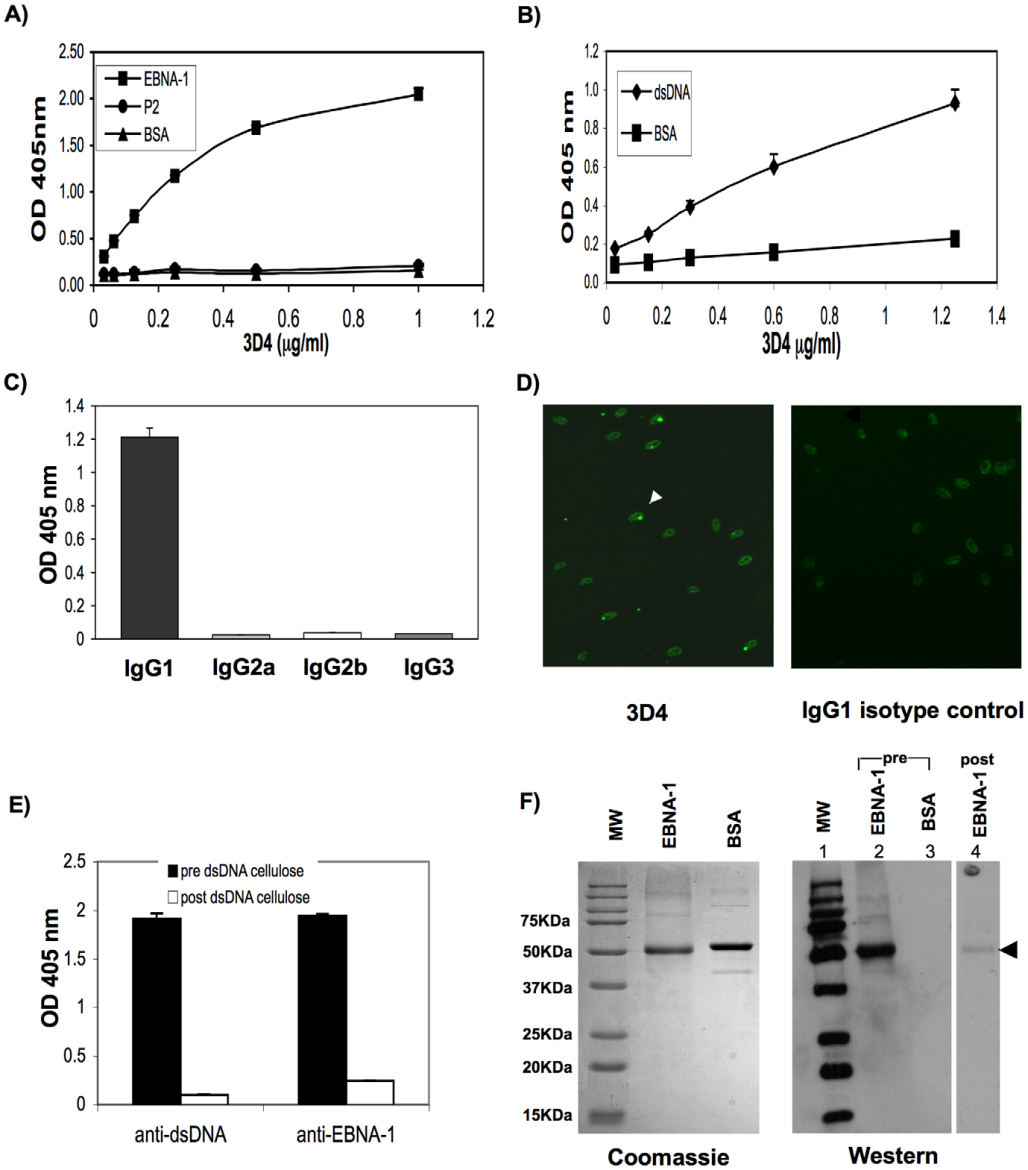
MAb, 3D4, is specific for both EBNA-1 and dsDNA. (A) Anti-EBNA-1 ELISA. 3D4 was tested by ELISA, at increasing concentrations, for binding to EBNA-1, BSA, and the cystovirus, polymerase protein, P2 (negative control) coated on Costar plates. Plates were read at 405 nm at 20 minutes. 3D4 shows specificity for EBNA-1 under these conditions. Results are the averages of triplicates and standard deviations are indicated. (B) 3D4 was tested by ELISA for binding to dsDNA coated on Immulon-2 plates. Plates were read at 405 nm at 1 hour. Results in A and B are the average of triplicates and standard deviations are indicated. (C) 3D4 antibody is of the IgG1 isotype. ELISA plates coated with unlabeled anti-IgG1, anti-IgG2a, anti-IgG2b, or anti-IgG3 were incubated with 1.5 µg/ml of 3D4 MAb followed by the respective polyclonal isotype specific antibodies conjugated to alkaline phosphatase. (D) Purified 3D4 at a concentration of 10 µg/ml was examined for binding to Crithidia luciliae slides. Left panel shows the binding of 3D4 to kinetoplasts of Crithidia luciliae (arrow). Right panel; IgG1 isotype control antibody, does not bind specifically to kinetoplasts. (E & F) 3D4 was adsorbed on a dsDNA cellulose column and pre and post adsorbed antibody were tested for binding to dsDNA and EBNA-1 by ELISA (E) and to EBNA-1 by Western blot (F). (E) 3D4 adsorbed on dsDNA cellulose beads was completely depleted of antibody with reactivity for dsDNA and EBNA-1 as detected by ELISA. Results represent OD 405 nm values after subtraction of non specific binding to cellulose only beads. Standard deviations of triplicate wells are indicated. Anti-dsDNA and anti-EBNA-1 ELISAs were performed on Immulon-2 and Costar plates respectively and ELISAs were developed when ODs on each plate reached maximal values. (F) Post dsDNA cellulose adsorbed 3D4 shows reduced binding to EBNA-1 by Western blot. Left panel: Coomassie stained polyacrylamide gel. Right panel: Western blot: filters were immunostained with pre (lanes 2 and 3) or post dsDNA cellulose adsorbed 3D4 MAb (lane 4). Molecular weight markers used in Western blot were conjugated to *strep-tag* and were detected with Strep-Tactin-HRP.

3D4 was isolated from hybridoma supernatant, on a protein G column. Following purification, 3D4 was shown to bind to EBNA-1 by ELISA even at concentrations as low as 0.125 µg/ml (Figure 3A). Specificity of this antibody was demonstrated by its lack of binding to a control viral antigen, cystovirus RNA polymerase, P2, isolated by Gottlieb et al [14] and BSA (Figure 3A).

Purified 3D4 was also shown to cross-react with dsDNA by ELISA (Figure 3B). 3D4 was shown by ELISA to be of the IgG1 isotype (Figure 3C). Reactivity of 3D4 with dsDNA was confirmed by its ability to recognize the dsDNA containing kinetoplasts of Crithidia luciliae (Figure 3D, left panel). An IgG1 isotype control MAb failed to bind kinetoplasts (Figure 3D, right panel). Cross-reactivity of 3D4 was further demonstrated by adsorption of the purified MAb over dsDNA cellulose beads and then testing pre and post adsorbed antibody for binding to dsDNA and EBNA-1 by ELISA and Western blot (Figure 3E and F). Adsorption over dsDNA cellulose resulted in complete depletion of 3D4 as detected by anti-dsDNA and and anti-EBNA-1 ELISAs (Figure 3E). Post dsDNA-cellulose adsorbed 3D4 antibody also showed dramatically reduced binding to EBNA-1 compared to pre-adsorbed 3D4, by Western blot (Figure 3F, right panel, compare lanes 2 and 4). No binding of pre-adsorbed 3D4 antibody to BSA was observed (lane 3).

**Figure 4:**
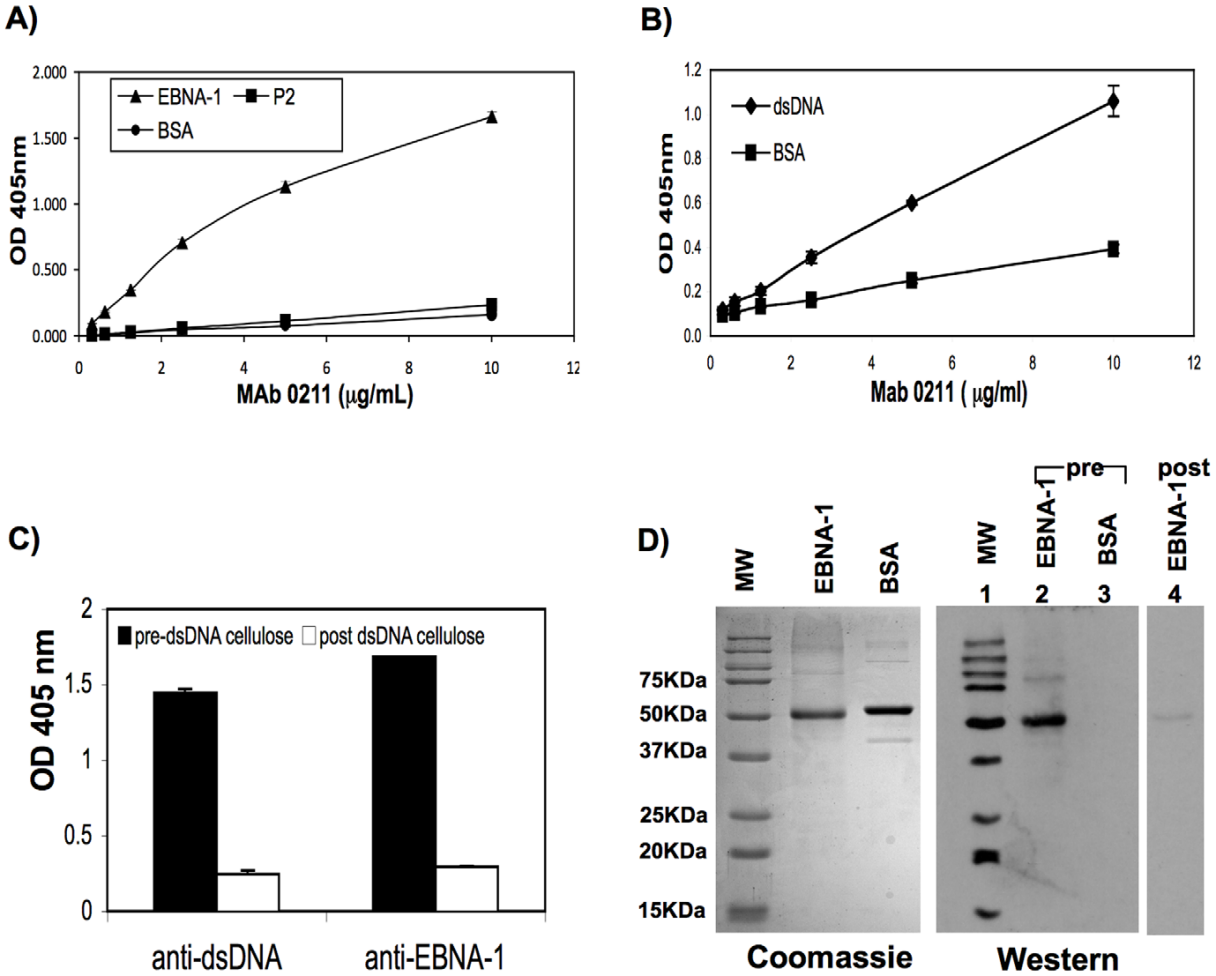
Commercial MAb, 0211 cross-reacts with dsDNA. (A) MAb 0211 binds to EBNA-1 as detected by ELISA but not P2, or BSA. (B) MAb 0211 binds to dsDNA as detected by ELISA. (C & D) MAb 0211 was adsorbed onto dsDNA cellulose beads and pre and post adsorbed antibody were tested for binding to dsDNA and EBNA-1 by ELISA. (C) MAb 0211 adsorbed on dsDNA cellulose beads, was completely depleted of antibody reactivity for dsDNA and EBNA-1. Anti-dsDNA and anti-EBNA-1 ELISAs were performed on separate ELISA plates and ELISAs were developed when ODs reached maximal values. Results represent OD 405 nm values after subtraction of non specific binding to cellulose only beads. (D) Post dsDNA cellulose adsorbed MAb 0211 shows reduced binding to rEBNA-1 by Western blot. Left panel: Coomassie stained polyacrylamide gel. Right panel: Western blot: filters were immunostained with pre (lanes 2 and 3) or post dsDNA cellulose adsorbed 0211 (lane 4) as indicated.

We were also interested in determining whether a monoclonal anti-EBNA-1antibody isolated from a completely different source would have similar binding properties to the 3D4 MAb isolated in our laboratory. We therefore examined the ability of a commercially prepared monoclonal IgG1 anti-EBNA-1 antibody, 0211 (Thermo Fisher Scientific/Pierce, Rockford, IL) to cross-react with dsDNA. The only information known about 0211 is that it was generated in response to EBNA-1, however, the exact epitope that it recognizes has not yet been identified. We first confirmed by ELISA, that this antibody binds to EBNA-1 but not to BSA or P2 (Figure 4A). We next observed by ELISA that this antibody also cross-reacts with dsDNA (Figure 4B). Furthermore, adsorption of 0211on a dsDNA cellulose column, resulted in complete depletion of the antibody as detected by anti- dsDNA and anti-EBNA-1 ELISAs (Figure 4C). A reduction in binding of post dsDNA cellulose adsorbed antibody to EBNA-1 was also demonstrated by Western blot (Figure 4D, right panel, compare lanes 2 and 4). No binding of pre-adsorbed 0211 antibody to BSA was observed (lane 3).

**Figure 5:**
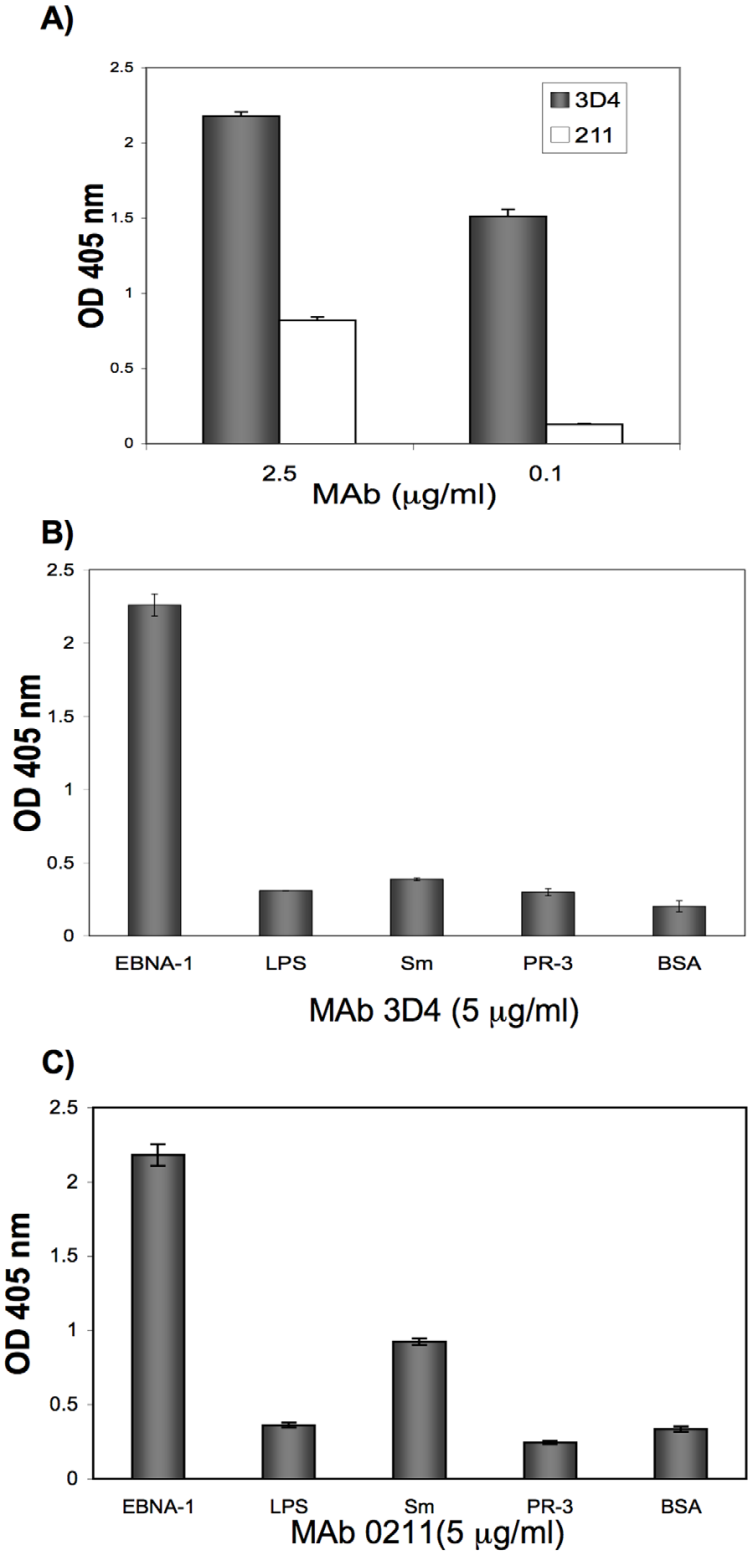
Comparison of the binding affinity and specificity of 3D4 and 0211. (A) Binding of 3D4 to EBNA-1 is compared to that of 0211 by ELISA at two different concentrations of MAbs. 3D4 binds more strongly to EBNA-1 than 0211. (B) Binding of 3D4 to several antigens is examined by ELISA. 3D4 does not show significant binding to other antigens tested. (C) Binding of 0211 to several antigens is examined by ELISA. 0211 binds moderately to Sm but not to the other antigens tested.

A comparison of MAbs 3D4 and 0211 revealed that although both antibodies bind strongly to EBNA-1, 3D4 has an even higher affinity for EBNA-1 than 0211 (Figure 5A). At a concentration of 0.1 µg/ml, 3D4 still bound robustly to EBNA-1 while binding by 0211 was negligible.

### MAbs 3D4 and 0211 were examined for binding to Sm and a panel of antigens

MAbs 3D4 and 0211 were also examined for binding to Sm, lipopolysaccharide (LPS), BSA, and proteinase -3 (PR-3) which is the target autoantigen in Wegener's granulomatosis. Antibodies to PR-3 are a subgroup of classic anti-neutrophil cytoplasmic antibodies (cANCA ). At 5 µg/ml, 3D4 displayed negligible binding to Sm relative to BSA (Figure 5B) while 0211 bound moderately well to Sm (Figure 5C). We also tested the binding of 3D4 and 0211 to LPS because it is negatively charged [15]. Since dsDNA is negatively charged, we wondered whether the MAbs would bind other negatively charged antigens. However, we observed that both 3D4 and 0211 failed to bind LPS.

### MAbs 3D4 and 0211 display differences in reactivity to the amino and carboxyl regions of EBNA-1

**Figure 6:**
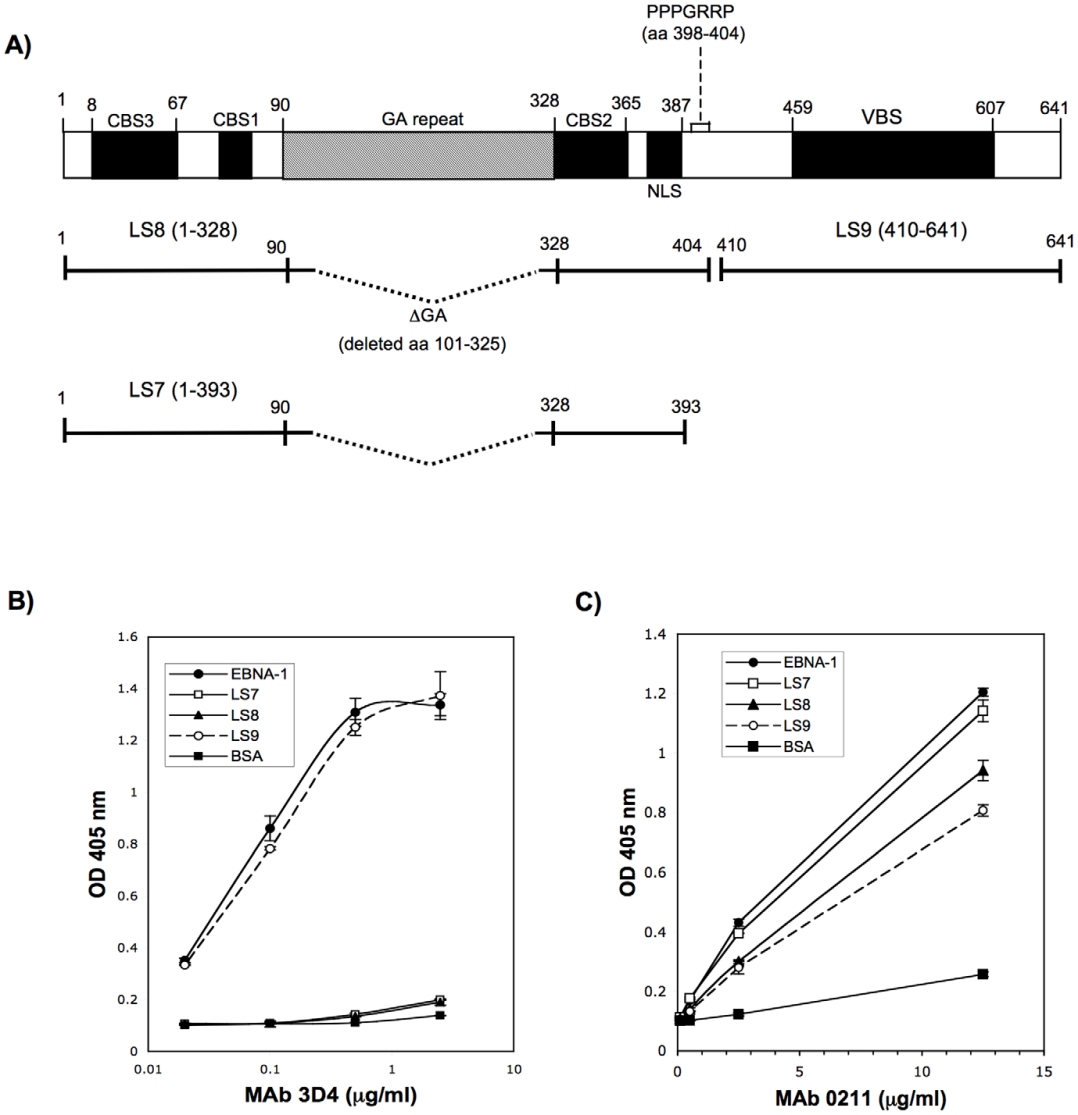
3D4 binds to the carboxyl region while 0211 binds to both the carboxyl and amino regions of EBNA-1. (A) Functional map of the complete EBNA-1 protein containing the Gly-Ala repeat region (GA). CBS 1,2,3; chromatin binding sites, NLS; nuclear localization site, VBS: viral binding site. LS8 denotes the amino fragment (aa 1–404) lacking most of the GA repeat. LS7 denotes the amino fragment (aa 1–393) lacking the GA repeat and lacking PPPGRRP. LS9 denotes the carboxyl fragment (aa 410–641). (B) MAb 3D4 is strongly reactive with LS9 but not LS8 or LS7 as detected by ELISA. (C) MAb 0211 is reactive with LS7, LS8 and LS9 as detected by ELISA. Results in B and C are the average of triplicate wells.

To begin to understand whether MAbs 3D4 and 0211 recognize the same or different regions of EBNA-1, they were examined by ELISA for binding to three truncated recombinant EBNA-1 proteins, LS7, LS8, and LS9, isolated in this laboratory from E. coli. These truncated recombinant proteins are comprised of the amino or carboxyl regions of EBNA-1. The protein designated LS8, is comprised of the amino region of rEBNA-1, from the initial Met residue to aa 404 (Figure 6A). Like rEBNA-1 used in this study, it lacks most of the Gly-Ala repeat. It contains the PPPGRPP region in EBNA-1 (aa 398–404) that was shown by James et al to be homologous to a proline rich epitope in Sm B/B′ [8]. LS7 is identical to LS8 except that it terminates at aa 393 and therefore lacks the PPPGRPP epitope (Figure 6A). The rational for generating two amino fragments, one with and one without the proline rich epitope was to determine whether this epitope which is responsible for eliciting cross-reactivity with Sm is also involved in eliciting cross-reactivity with dsDNA. LS9 comprises the carboxyl region of the rEBNA-1 protein from aa 410 to the terminal aa 641 and lacks the proline epitope (Figure 6A). MAb 3D4 was observed to bind strongly to LS9 but not at all to LS7 or LS8 (Figure 6B). The kinetics of 3D4 binding to LS9 closely paralleled the kinetics of binding to the entire rEBNA-1 protein indicating that this carboxyl region (aa 410–641) is sufficient for optimal recognition by 3D4. Adsorption of 3D4 to dsDNA cellulose was also observed to remove all binding to the carboxyl fragment. Taken together these results suggest that the cross-reactive epitope recognized by 3D4, is configured within the carboxyl region.

MAb 0211 was observed to bind all three truncated proteins indicating that it recognizes epitopes in both the amino and carboxyl regions of EBNA-1, however the binding to the amino proteins, LS7 and LS8 is better than the binding to the carboxyl protein, LS9 (Figure 6C). Interestingly, 0211 binds more strongly to LS7 than LS8 and since LS7 does not contain the PPPGRPP epitope, this indicates that the proline epitope is not necessary for the binding of 0211 to EBNA-1. Furthermore, this proline rich region may structurally interfere with binding by 0211. It cannot be determined at this time whether the epitope in the amino or carboxyl region of EBNA-1 is responsible for MAb 0211's cross-reactivity with dsDNA.

Despite the fact that 3D4 and 0211 bind differently to the amino and carboxyl regions of EBNA-1, both antibodies still cross-react with dsDNA. Consequently there could be more than one EBNA-1 epitope that could be linear or conformational, that acts as a mimotope for dsDNA. Alternatively, the epitope (s) in the carboxyl region may be more important for cross-reactivity with dsDNA and since 0211 also binds Sm, the epitope in the amino region may be more important for cross-reactivity with Sm.

### 3D4 binds to a 148 aa core domain in the carboxyl region of EBNA-1 that lacks the negatively charged C-terminal amino acids

**Figure 7:**
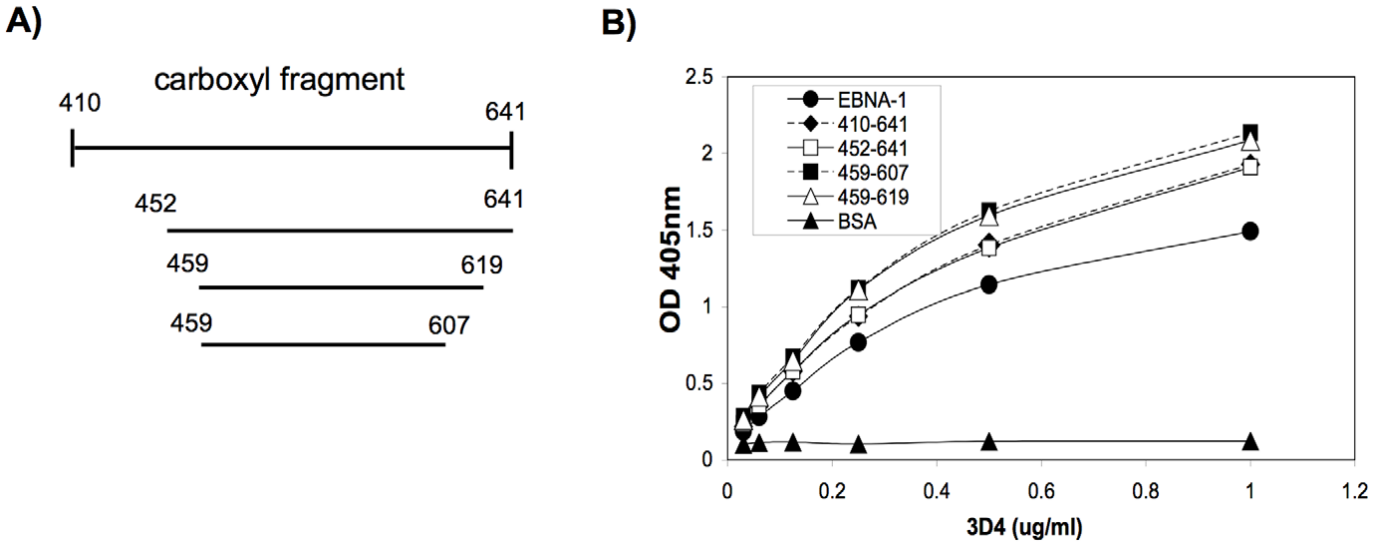
3D4 recognizes a 148 aa core domain in the carboxyl region of EBNA-1. (A) Map of the carboxyl region of EBNA-1 and 3 truncated carboxyl fragments. (B) 3D4 was tested by ELISA for binding to the 3 truncated fragments of EBNA-1. 3D4 binds strongly to all 3 fragments, the smallest being EBNA_459–607_.

To begin to identify a smaller fragment in the carboxyl region of EBNA-1 that contains the epitope recognized by 3D4, we examined the binding of this MAb to three truncated carboxyl fragments; EBNA_452–641_, EBNA_459–619_, and EBNA_459–607_ (Figure 7A). These fragments are expressed by plasmids kindly provided to us by Dr. Lori Frappier [16]. We observed that 3D4 bound all 3 fragments equally well and did not show diminished binding to these fragments relative to the entire carboxyl region (EBNA_410–641_) (Figure 7B). In fact 3D4 displayed optimal binding to the smallest fragment, EBNA_459–607_ suggesting that the cross-reactive epitope lies within this 148 aa region. The carboxyl region of EBNA-1 has a net negative charge due to the high frequency of negatively charged amino acids at the C-terminus (aa 619–641). Twelve out of 22 of the C terminal amino acids are either glutamic or aspartic acid. Both, EBNA_459–619_, and EBNA_459–607_ lack these negatively charged amino acids. Since removal of these negatively charged amino acids did not diminish recognition by 3D4, this suggests that charge interaction is not the basis for 3D4's binding to EBNA-1. MAb, 0211 displays a similar binding pattern to the truncated carboxyl fragments of EBNA-1 with maximal binding to the two smallest fragments EBNA_459–619_ and EBNA_459–607_ (data not shown).

## Discussion

This study demonstrates for the first time, that some antibodies that arise in response to EBNA-1 cross-react with dsDNA. Our laboratory previously demonstrated that EBNA-1 expression could elicit an anti-dsDNA response, however, it was not known at the time whether the antibodies to dsDNA were distinct from the anti-EBNA-1 antibodies or whether the same antibodies that bound EBNA-1 were also able to recognize dsDNA. The demonstration that purified monoclonal IgG antibodies to EBNA-1 also bind dsDNA and that adsorption of these antibodies on a dsDNA cellulose column, removes EBNA-1 reactivity, confirms the cross-reactive nature of these antibodies. However, this does not exclude the potential role of epitope spreading in the development of the cross-reactive response. The delay in the development of a strong anti-dsDNA response relative to the anti-EBNA-1 response suggests that cross-reactivity continues to develop over time. It may be that early in the response to EBNA-1, the epitopes that are targeted, elicit only a weakly cross-reactive response to dsDNA. Later in the response, other epitopes may be targeted as a result of intra-molecular epitope spreading and these latter epitopes may be the ones that are responsible for cross-reactivity with dsDNA. Alternatively, antibodies that cross-react more strongly with dsDNA may arise later in the immune response as a consequence of somatic mutation. A specific mutation in the variable heavy and/or light chain regions of an anti-EBNA-1 antibody may alter its specificity from one that only recognizes EBNA-1 to one that recognizes EBNA-1 as well as dsDNA.

While all mice injected with EBNA-1 developed antibodies to dsDNA, we did not consistently observe features of clinical lupus in these mice. Two out of 5 injected mice had significant levels of protein but no blood in their urine relative to uninjected mice. The kidney of 1 out of 3 mice examined at 3 months post injection, had evidence of some IgG immune complex deposition, however, none of the kidneys examined showed signs of lupus histopathology (data not shown). Future studies will include examining a larger cohort of mice for evidence of glomerulonephritis and investigating whether 3D4 and 0211 can deposit in the kidney.

Antibodies to a variety of self proteins have been reported to cross-react with dsDNA, such as antibodies to extracellular matrix protein, HP8, ribosomal P protein, elongation factor-2 (EF-2), α-actinin, the NMDA receptor and Sm D [17], [18], [19], [20], [21], [22]. Antibodies targeting peptide mimotopes of dsDNA such as DWEYSVWLSN and RLTSSLRYNP have also been reported [23], [24]. In addition, antibodies to microbial antigens such as glycolipid components of the cell wall of Mycobacterium tuberculosis, phosphorylcholine in the cell wall of Streptococcus pneumoniae or proteins in Burkholderia fungorum have been observed to cross-react with dsDNA [25], [26], [27], [28]. It is unclear how these antigens act as molecular mimics to dsDNA, but it may be due to similarities in the 3 dimensional structures of these antigens and dsDNA. Evidence from some studies suggest that conformational epitopes are the targets of antibodies that cross-react with dsDNA and self proteins [29], [30].

Most of the monoclonal anti-EBNA-1 antibodies generated in this study were found to cross-react with dsDNA. Very few of them were found to recognize EBNA-1 only. This may be because mice that were selected for fusion had already developed maximal levels of cross-reactive antibodies either due to epitope spreading or somatic mutation. In addition, the rEBNA-1 protein used in our injection studies, lacks the Gly-Ala repeat which has been shown to be a major epitope that elicits anti-EBNA-1 antibodies in normal individuals [31]. It may be that in the absence of the Gly-Ala repeat, the response is biased towards other epitopes some of which happen to elicit antibodies that also cross-react with dsDNA. It was previously demonstrated that patients with lupus tend to mount an immune response to different epitopes on EBNA-1 than healthy individuals [4], [31], [32]. While sera from healthy individuals, preferentially react with the Gly-Ala repeat, sera from lupus patients tend to recognize epitopes in the amino and carboxyl terminal regions of EBNA-1 that are more likely to be cross-reactive with nuclear autoantigens. It is not clear whether lupus patients are genetically predisposed to developing cross-reactive antibodies or whether they have a defect in B cell tolerance leading to failed regulation of the autoreactive B cells producing these antibodies.

The two MAbs that were extensively characterized in this study, 3D4 and 0211, bind to EBNA-1 and dsDNA, yet 3D4 recognizes only the carboxyl region in EBNA-1, while 0211 recognizes both the amino and carboxyl regions. A homology search failed to find any region in EBNA-1 that is homologous to two previously described peptide mimotopes for dsDNA; DWEYSVWLSN and RLTSSLRYNP. It is not yet known whether 3D4 and 0211 recognize a homologous epitope in the carboxyl region, however the C-terminal negatively charged amino acids do not appear to be necessary for the binding of either of these MAbs to the carboxyl region.

MAb 0211 binds moderately well to Sm while 3D4 displays negligible binding to Sm. The basis for the cross-reactivity of 0211 with Sm does not seem to be dependent on the proline rich epitope described by James et al, since 0211 binds even stronger to a truncated amino fragment of EBNA-1 lacking this determinant [8]. It is not yet clear whether the epitopes in the amino and carboxyl region recognized by 0211 share homology. However, the observation that 0211 binds to both Sm and the amino fragment, while 3D4 binds to the carboxyl region only, suggests that an epitope in the amino fragment may be more important for cross-reactivity with Sm while an epitope in the carboxyl region may be important for cross-reactivity with dsDNA. In a preliminary study that is consistent with this, we recently identified a monoclonal IgM antibody that reacts strongly with EBNA-1 and dsDNA but not Sm and only recognizes the carboxyl region of EBNA-1. In addition, recent studies reveal that the MAb, 9G3 (Table 1) that recognizes BSA, also cross-reacts with Sm, and binds to both the amino and carboxyl fragments of EBNA-1 (preliminary data). The polyreactive nature of this antibody, which will be examined in more depth in future studies, is potentially important since polyreactive antibodies have been shown to be the precursors of more pathogenic antibodies in lupus [33].

As previously mentioned, the basis for antibody cross-reactivity with EBNA-1 and dsDNA does not appear to be charge interactions since removal of the negatively charged amino acids from the carboxyl region of EBNA-1 does not diminish binding to this region (Figure 7). Furthermore, neither 3D4 nor 0211 recognize LPS which is negatively charged (Figure 5). It is possible that the epitopes in EBNA-1 that cross-react with dsDNA are structural. The X-ray structure of the crystallized VBS/DNA binding region of EBNA-1 has been determined and reveals two distinct domains; a core domain that mediates protein dimerization and a flanking domain that mediates base contact with dsDNA [34]. These domains possess much secondary structure, which may serve as targets for antibodies that cross-react with dsDNA. The core domain (aa 504–607) contains a ß sheet, an α helix and a proline loop and the flanking domain (aa 470–503) contains α helixes. Potentially, the cross-reactive antibodies may recognize a portion of the α helix that mimics the α helix in dsDNA. The X-ray crystal structure of the N-terminus has not yet been resolved. However, this region appears to be less structured than the carboxyl region and could be flexible enough to fold onto itself or the carboxyl region providing multiple opportunities for antibody interaction.

It will be important in future studies to map the epitopes in EBNA-1 that lead to the cross-reactivity with dsDNA and determine whether or not these epitopes are conformational. Identifying these epitopes and individuals who preferentially produce antibody responses to them, may be useful for determining those who are at risk of developing lupus so that early treatment strategies can be initiated. In addition, knowledge of these epitopes may help in the design of therapeutic strategies that can mask these epitopes thereby preventing the immune system from mounting a cross-reactive response to them.

## Materials and Methods

All animals were handled in strict accordance with good animal practice as defined by federal and state policies set forth by The Public Health Service Policy on the Humane Care and Use of Laboratory Animals (PHS 1986), The Guide for the Care and Use of Laboratory Animals (ILAR 1996), and The USDA Animal Welfare Act (CFR 1985). All work done with animals in this study, was approved by The Institutional Animal Care and Use Committee (IACUC) at The City College of New York, (approval numbers 626 and 828).

### Extraction, Purification and Characterization of rEBNA-1 lacking the Gly-Ala repeat

The EBNA-1 baculovirus expression vector used in this study was a generous gift from Dr. Lori Frappier (McMaster University, Ontario, Canada). Recombinant EBNA-1 protein (rEBNA-1) was isolated from this baculovirus expression vector according to Lori Frappier (personal communication and modifications of Frappier and O'Donnell) [35]. This vector encodes an EBNA-1 protein that has a deletion of most of the Gly-Ala repeat and has a 6× His tag on the N-terminus, which allows for the protein's isolation on a Ni^2+^ metal affinity column. Briefly, SF9 cells were grown in serum-free insect cell culture medium, Sf-900 II SFM (Invitrogen, Carlsbad, CA) at 27°C. Cells were resuspended at a concentration of 1×10^6^ cells per ml and 100 ml of cells (100×10^6^ cells total) were infected with 500 µl of high titer recombinant EBNA-1 baculovirus and grown in 500 ml Erlenmeyer flasks (Corning, Acton, MA) at 27°C in an air shaker for 60 hours. The cells were then harvested by centrifuging at 2000 rpm at 4°C for 10 minutes. The cell pellets were resuspended in 25 ml of a hypotonic buffer (20 mM HEPES pH 7.8, 1 mM MgCl_2_, 1 mM PMSF and 10 µM leupeptin) and allowed to swell on ice for 10 minutes. Cells were then dounced 20 times on ice and centrifuged at 4°C at 3000 rpm for 10 minutes. Supernatant was discarded. The pellet containing intact nuclei was resuspended in 25 ml of hypotonic buffer containing 2.7 ml of 5 M NaCl. After douncing on ice to open the nuclear envelope, the fraction was centrifuged at 18,000 rpm for 20 minutes and the supernatant containing rEBNA-1 protein was collected. Further purification of rEBNA-1 was performed employing a nickel agarose (Ni^2+^-NTA) (QIAGEN, Valencia, CA) column according to modifications of Ceccarelli and Frappier [12]. Ni^2+^-NTA agarose (1ml) was equilibrated in column buffer (0.2 M Hepes pH 7.8, 0.5 M NaCl, 10% glycerol) containing 5 mM imidazole, at room temperature. The nuclear extract was incubated with pre-equilibrated Ni^2+^-NTA at room temperature for 2 hours, with rocking. After incubation, a column was packed with the nuclear extract/Ni^2+^-NTA slurry. The column was washed slowly with column buffer containing 5 mM imidazole followed by column buffer containing 25 mM imidazole. Next, the EBNA-1 protein was eluted with column buffer containing 300 mM imidazole. The protein was then concentrated and the buffer exchanged with PBS, 250 mM NaCl using an Amicon Centrifugal filter (10,000 molecular weight cut off) (Millipore, Billerica, MA). The protein was then resolved by 12% SDS-PAGE followed by a Western blot and immunostaining with a monoclonal antibody to EBNA-1.

### Injection of mice with rEBNA-1 protein

Fifteen, six week old, female BALB/c mice were used for injection studies. Five mice were injected intraperitoneally (ip) with 50 µg of rEBNA-1 protein in complete Freund's adjuvant (CFA) (Sigma, St Louis, MO) in a 1∶1 (v/v) ratio and boosted twice (at weeks 3 and 9) with 25 µg of rEBNA-1 in incomplete Freund's adjuvant (IFA). Five mice were injected with CFA only and boosted with IFA and 5 age-matched control mice remained uninjected throughout the study. The mice were bled immediately before injection and at weeks 1.5, 4, 6, 10, 12, 15 and 18. The sera obtained, from these mice were tested for anti-EBNA-1 and anti-dsDNA antibodies by ELISA.

### Construction of plasmids encoding the amino and carboxyl regions of EBNA-1

Truncated EBNA-1 proteins were isolated from plasmid transformed E. coli cells. The pLS8 expression plasmid carries the encoding sequence for the amino terminus of the EBNA-1 antigen, from the initial Met residue to amino acid position 404 and lacks virtually all of the Gly-Ala repeat. It was prepared by PCR amplification of the EBNA-1 gene from pMRC72 [10] which contains the EBNA-1 coding sequence, but lacks the Gly-Ala repeat, using the following primer pair; EBV7, 5 – CATATGTCTGACGAGGGGC CAGGT-3′ (forward primer) and EBV6, 5′-CTCGAGTTATGGCCTTCTACCTGG-3′ (reverse primer). The pLS7 expression plasmid also carries the encoding sequence for the amino terminus of the EBNA-1 protein, from the initial Met residue but it terminates at amino acid position 393. Like pLS8, it lacks most of the Gly-Ala repeat. However, unlike pLS8, it is missing the PPPGRRP epitope (aa 398–404). It was prepared from pMRC72 using the following primer pair; EBV7 (see above) and EBV5, 5′ CTCGAGTTAAGACCCGGAT GATGA 3′ (reverse primer). The pLS9 expression plasmid carries the EBNA-1 encoding sequence for the carboxyl terminus of EBNA-1 from amino acids 410 to 641. It was also prepared by PCR amplification of the EBNA-1 gene from pMRC72 using the following primer pair; EBV3, 5′-CATATGGGGGAA GCCGATTA TTTTGAAT-3′ (forward primer) and EBV 4, 5′-CTCGAGTTACTCCTGCCCTTCCTC-3′ (reverse primer). The PCR amplifications were performed for 30 cycles. The amino and carboxyl PCR fragments were digested with Nde1 and Xho1 and inserted into the pET28A expression vector (Novagen, San Diego, CA) which contains an N-terminal 6× His tag.

### Isolation of truncated recombinant EBNA-1 proteins

E. coli colonies transformed with pLS7, pLS8, or pLS9 (see above) were selected on LB ampicillin plates and grown at 37°C in 50 ml of LB media containing 1% glucose. Cultures were diluted in 490 ml LB with 0.1 mM IPTG and grown for several hours at 20°C to a final OD_600_ of approximately 0.6. Cultures were harvested and re-suspended in lysis buffer (50 mM Tris-Hcl, pH 7.8, 250 mM NaCl) containing 1.0 mM PMSF. Cells were sonicated for 15 minutes on ice with a 4 second on pulse, 6 seconds off at a 30% amplitude. The cell lysate was cleared by centrifugation at 10,000 rpm for 30 minutes at 4°C and filtered through a 45 µM filter. Five mls of Ni^2+^-NTA beads equilibrated with lysis buffer were added to the cleared supernatant and incubated with gentle rocking at room temperature. The beads (bound to the recombinant protein) were separated from the supernatant by low- speed centrifugation. They were then washed 6 times with wash buffer (50 mM Tris-HCl, ph 7.8, 250 mM Nacl, 60 mM imidazol, and 10% glycerol). Two ml of elution buffer (50 mM Tris-Hcl, pH 7.8, 250 mM NaCl, 250 mM imidazol, 10% glycerol) were added to the beads and beads were rocked for 15 minutes. The beads were removed from the reaction by low-speed centrifugation. Supernatants containing the recombinant protein were concentrated and the buffer was exchanged with PBS, 250 mM NaCl using an Amicon Centrifugal filter. Proteins were analyzed by SDS-PAGE and Western Blot.

Plasmids (vector pET15b) expressing the following EBNA-1 amino acid sequences; EBNA_452–641_, EBNA_459–607_, and EBNA_459–619_ were gifts from Dr. Lori Frappier [16]. Soluble truncated EBNA-1 proteins were produced in Escherichia coli strain BL21 (DE3) and isolated from cell-lysates. Proteins were then purified over a Ni-NTA agarose column as described above. Proteins were analyzed by SDS-PAGE and Western Blot.

### ELISAs

#### Detection of antibodies to EBNA-1, dsDNA, Sm, LPS, Proteinase 3 and BSA

Diluted serum samples from EBNA-1 injected mice, hybridoma supernatants, or purified monoclonal antibodies were tested for binding to EBNA-1, dsDNA, Sm, LPS, or PR-3 by ELISA as previously described [10], [36]. For the detection of antibodies to EBNA-1, LPS, Proteinase 3, and BSA, Costar plates (Corning Incorporated, Corning, NY) were coated in PBS with 2.0–5.0 µg/ml of antigen. Costar plates were coated overnight with 5.0 µg/ml of Sm (Immunovision, Springdale, AR) in 0.1M carbonate buffer for the detection of antibodies to Sm. For the detection of antibodies to dsDNA, Immulon-2 plates (Dynatech Laboratories, Inc., Chantilly, VA) were coated with 100 µg/ml of calf thymus dsDNA.

#### Detection of antibody binding to truncated amino or carboxyl fragments of EBNA-1

Purified monoclonal antibodies were tested for binding to truncated amino (LS7 and LS8) and carboxyl regions (LS9, EBNA_452–641_, EBNA_459–619_, and EBNA_459–607_) of EBNA-1. ELISA plates were coated with 2.0 µg/ml of the purified, truncated recombinant proteins isolated in this laboratory. Subsequent steps in the ELISA were performed according to Sundar et al [10].

#### Isotype ELISA

ELISA plates were coated with 50 µl of a 1∶1000 dilution of either unlabeled goat anti-mouse IgG1, IgG2a, IgG2b or IgG3 (Southern Biotech, Birmingham, Alabama) and incubated at 37°C for one hour and overnight at 4°C. Monoclonal 3D4 antibody was diluted to 1.5 µg/ml and incubated on the plate for one hour at 37°C. Next, 50 µl of a 1∶1000 dilution of goat anti-mouse IgG1 conjugated to alkaline phosphatase (AP), anti-IgG2a-AP, anti-IgG2b-AP, or anti-IgG3-AP (Southern Biotech) was added to wells coated with unlabeled anti-IgG1, anti-IgG2a, anti-IgG2b, or anti- IgG3 respectively. Color development was measured following the addition of 4-nitrophenyl-phosphate disodium salt as substrate and plates were read at 405 nm on a Titertek Multiscan ELISA plate reader.

#### Quantitative ELISA

A quantitative ELISA was performed, as previously described, to determine the concentration of purified monoclonal IgG antibodies in hybridoma supernatants [36]. Briefly, ELISA plates were coated overnight with 1.0 µg/well of goat anti-mouse IgG antibody (Southern Biotechnology). A commercial mouse monoclonal IgG antibody (Sigma) was serially diluted, beginning at a concentration of 200 ng/ml and used to generate a standard curve. Serial dilutions of monoclonal antibody purified in this laboratory were applied to the anti-IgG coated wells and the concentration of antibody was extrapolated from the standard curve. Monoclonal antibodies were detected with goat anti-mouse IgG antibody conjugated to AP followed by the addition of 4-nitrophenyl-phosphate disodium salt as substrate.

### Crithidia Assay

Ready to use Crithidia slides from the CrithiDNA Anti-nDNA Antibody Test Kit from Antibodies Inc. (Davis, CA), were immunostained either with mouse sera from EBNA-1 injected mice, diluted 1/50 or with purified monoclonal antibody diluted to 10 µg/ml. Slides were incubated in a moist, dark chamber for 30 minutes at room temperature (RT). A positive control anti-dsDNA antibody was provided with the kit. A nonspecific monoclonal mouse IgG1 antibody was used as an isotype control (Sigma). Next, the slides were extensively washed with PBS and immunostained for 30 minutes at RT with a 1∶250 dilution of biotinylated goat anti mouse IgG (Southern Biotech). This was followed by 20 µl of a 1∶500 dilution of Streptavidin-FITC (Southern Biotech) for 30 minutes at RT. Slides were washed again and Prolong Gold Antifade, (Invitrogen, Carlsbad, CA) was added prior to examination by fluorescence microscopy using a Nikon Eclipse microscope, model, TE 2000-S at a magnification of 400×.

### Western Blot

Proteins were analyzed by SDS-PAGE on a 12% gel and transferred to a nitrocellulose membrane using a Bio-Rad wet transfer apparatus (BioRad, Hercules, CA). After transfer, the membranes were blocked with 3% Milk-PBS for one hour at RT with shaking. The blot was incubated overnight at 4°C with a MAb generated in our laboratory (3D4), diluted to 1 µg/ml or a commercially prepared MAb, 0211 (Thermo Fisher Scientific/Pierce, Rockford, IL) diluted to 10 µg/ml according to the manufacturers protocol. The membrane was washed 6 times in wash buffer (PBS, 0.05% Tween-20). Bound MAbs antibodies were detected with HRP-conjugated goat anti mouse IgG (Southern Biotech) diluted 1∶20,000, followed by chemiluminescence using the Pierce ECL kit according to the manufacturers protocol (Pierce, Rockford, IL). Molecular weight markers conjugated to *strep-tag* (Precision plus protein WesternC) (Biorad, Hercules, CA) were detected with a 1∶20,000 dilution of Strep-Tactin-HRP (Biorad).

### Somatic Cell Fusion

BALB/c mice were immunized intraperitoneally (ip) with 50 µg/ml of rEBNA-1 in CFA and then boosted at 3, 7, and 12 weeks with 25 µg/ml of rEBNA-1 in IFA. Three to four days following the third boost, splenocytes were fused with NSO cells according to Iliev et al [37]. They were grown in complete HAT media supplemented with 20% FBS, 10% NCTC, 1% Penicillin-Streptomycin, 1% non-essential amino acids and 1% L-glutamine. Supernatants from hybridomas were tested for IgG anti-EBNA-1 and anti-dsDNA antibodies by ELISAs as described above.

### Purification of Monoclonal Antibodies

Hybridomas producing a MAb to EBNA-1 were grown in serum free media (Hyclone, Logan, Utah) and 400 ml of supernatant were collected for IgG purification. Antibody was purified from the supernatant by eluting it off a protein G Sepharose column (Gamma Bind™ Plus Sepharose ™ gel beads, Amersham Pharmacia, Uppsala, Sweden) with 0.1M glycine pH 2.5, according to the manufacturer's protocol. Column eluate was neutralized with 1M Tris-HCl. The purified antibody was dialyzed overnight with PBS and antibody concentration was determined by a quantitative ELISA (above).

### Antibody adsorption on dsDNA-cellulose columns

Columns were packed with 0.5ml of calf thymus dsDNA-cellulose or cellulose beads (Sigma, St.Louis, MO) according to the manufacturer's protocol. The columns were washed with 10 mM Tris buffer pH 7.9 containing 1 mM EDTA. Columns were then blocked with 5% FBS-PBS overnight at 4°C. A 1/1,000 dilution of week 4 and a 1/5000 dilution of week 12, rEBNA-1 injected mouse sera or 5 µg/ml of MAbs, 3D4 or 0211 were slowly loaded onto cellulose and dsDNA cellulose columns and allowed to sit for 1 hour at 4°C. The flow through was collected and pre and post adsorbed sera or monoclonal antibody were tested for binding to dsDNA and EBNA-1 by ELISA and Western blot as described above.
